# Epistatic Roles of E2 Glycoprotein Mutations in Adaption of Chikungunya Virus to *Aedes Albopictus* and *Ae. Aegypti* Mosquitoes

**DOI:** 10.1371/journal.pone.0006835

**Published:** 2009-08-31

**Authors:** Konstantin A. Tsetsarkin, Charles E. McGee, Sara M. Volk, Dana L. Vanlandingham, Scott C. Weaver, Stephen Higgs

**Affiliations:** Department of Pathology, University of Texas Medical Branch, Galveston, Texas, United States of America; Institut Pasteur, France

## Abstract

Between 2005 and 2007 Chikungunya virus (CHIKV) caused its largest outbreak/epidemic in documented history. An unusual feature of this epidemic is the involvement of *Ae. albopictus* as a principal vector. Previously we have demonstrated that a single mutation E1-A226V significantly changed the ability of the virus to infect and be transmitted by this vector when expressed in the background of well characterized CHIKV strains LR2006 OPY1 and 37997. However, in the current study we demonstrate that introduction of the E1-A226V mutation into the background of an infectious clone derived from the Ag41855 strain (isolated in Uganda in 1982) does not significantly increase infectivity for *Ae. albopictus*. In order to elucidate the genetic determinants that affect CHIKV sensitivity to the E1-A226V mutation in *Ae. albopictus*, the genomes of the LR2006 OPY1 and Ag41855 strains were used for construction of chimeric viruses and viruses with a specific combination of point mutations at selected positions. Based upon the midgut infection rates of the derived viruses in *Ae. albopictus* and *Ae. aegypti* mosquitoes, a critical role of the mutations at positions E2-60 and E2-211 on vector infection was revealed. The E2-G60D mutation was an important determinant of CHIKV infectivity for both *Ae. albopictus* and *Ae. aegypti*, but only moderately modulated the effect of the E1-A226V mutation in *Ae. albopictus*. However, the effect of the E2-I211T mutation with respect to mosquito infections was much more specific, strongly modifying the effect of the E1-A226V mutation in *Ae. albopictus*. In contrast, CHIKV infectivity for *Ae. aegypti* was not influenced by the E2-1211T mutation. The occurrence of the E2-60G and E2-211I residues among CHIKV isolates was analyzed, revealing a high prevalence of E2-211I among strains belonging to the Eastern/Central/South African (ECSA) clade. This suggests that the E2-211I might be important for adaptation of CHIKV to some particular conditions prevalent in areas occupied by ECSA stains. These newly described determinants of CHIKV mosquito infectivity for *Ae. albopictus* and *Ae. aegypti* are of particular importance for studies aimed at the investigation of the detailed mechanisms of CHIKV adaptations to its vector species.

## Introduction

The recent massive epidemics of Chikungunya virus (CHIKV) in Africa, the Indian Ocean islands, India, and the small outbreak in Europe have elevated this arthropod-borne virus (arbovirus) to the status of a major global health problem [1]. CHIKV, a member of the *Alphavirus* genus in family *Togaviridae*, is transmitted to humans by *Aedes* (*Stegomyia*) spp mosquitoes, primarily *Ae. aegypti*. However, transmission by a previously unrecognized vector species, *Ae. albopictus*, has been a critical contributor facilitating recent epidemics [2]–[8].

Phylogenetic analysis of CHIKV strains obtained during outbreaks circulating in *Ae. albopictus*-human transmission cycles have identified the independent acquisition of a common mutation, namely E1-A226V, in strains isolated from different geographic regions [2], [5], suggesting that this mutation is associated with specific genetic adaptations to *Ae. albopictus* mosquitoes. Recently we demonstrated that the E1-A226V mutation significantly increases the ability of CHIKV to infect and be transmitted by a laboratory colony of *Ae. albopictus* mosquitoes when expressed in the background of the well-characterized La Reunion LR2006 OPY1 and West-African 37997 CHIKV strains [9]. Furthermore, CHIKV isolates from Reunion Island possessing valine at position E1-226 disseminate significantly more efficiently to the salivary glands of *Ae. albopictus* mosquitoes collected from La Reunion Island and Mayotte, as compared with CHIKV isolates bearing alanine at this position [10]. Taken together, these findings provide compelling evidence that the E1-A226V mutation is a major genetic determinant of adaptation of CHIKV to a new vector species, *Ae. albopictus*, and provides a plausible explanation for how this mutant CHIKV caused an epidemic in a region lacking the more typical urban vector, *Ae. aegypti*.

Alphaviruses are enveloped single-stranded positive-sense RNA viruses. Genomic RNA, of ∼12,000 nt, consists of two open reading frames (ORF): the first, translated from genomic RNA, encodes the four non-structural proteins (nsP1–4), whilst the second ORF is translated from sub-genomic RNA to produce the three main structural proteins (capsid, E2 and E1). The E2 and E1 envelope glycoproteins form heterodimers on the viral surface, where E2 lies mainly on top of E1 and is believed to interact with cellular receptors [11]. Following binding and endocytosis, the E1 underlying E2 mediates fusion of viral and cellular membranes within the acidic conditions of endosomal compartments [12].

*Ae. albopictus* mosquitoes are native to Southeast Asia, but have recently spread globally due to the advent of modern shipment, with the current geographic range including Europe, Africa, the Middle East, North and South America and the Caribbean [13], [14]. As a consequence of this recent range expansion, the pathogens transmitted by this species may be introduced or reemerge in new areas. This scenario was exemplified in August and September of 2007, when the CHIKV–*Ae. albopictus* transmission cycle was established for the first time in Europe, with an estimated 254 human cases in Italy [4], [6], [15], [16]. Besides CHIKV and dengue virus, *Ae. albopictus* has been demonstrated to be susceptible to infection by several clinically important arboviruses including; eastern [17], [18], and Venezuelan equine encephalitis (VEEV) [19], [20], yellow fever [21], West Nile [22]–[24], Japanese encephalitis [25], and Rift Valley fever viruses [26], among others. Understanding the mechanism(s) responsible for adaptation of arboviruses to a new vector may enhance our ability to predict spatial and temporal epidemic risks, and to direct vector control efforts towards specific arthropods, and so will enhance our ability to reduce the incidence of these diseases.

Previous investigations of the effects of the E1-A226V mutation on CHIKV infection of midguts, dissemination into salivary glands, and transmission to a vertebrate host by *Ae. albopictus* suggested that the epidemiologic success of CHIKV with the E1-A226V mutation was most likely due to enhanced midgut infectivity [9], [10]. The ability of CHIKV with A or V residues in position E1-226 to disseminate to salivary glands and be transmitted to suckling mice by orally infected and intrathoracically injected *Ae. albopictus* was also compared. When intrathoracically injected into the mosquito hemocoel, CHIKV does not need to infect midgut cells and can directly infect secondary organs including the salivary glands. Since in intrathoracically infected *Ae. albopictus* the E1-A226V mutation did not enhance dissemination/transmission rates (Higgs lab unpublished data), it was thus concluded that the effect of this mutation occurs before virus is released from the midgut into the hemocoel. These data, together with previous findings [9], [10], support the hypothesis that increased *Ae. albopictus* midgut infectivity resulting from the A226V mutation plays a primary role in enhanced viral transmission.

Little is known about the molecular mechanisms responsible for the selective advantage associated with the E1-A226V mutation. Earlier we demonstrated that the E1-A226V mutation was responsible for modulation of the CHIKV cholesterol dependence for replication in C6/36 cells, suggesting that specific lipid composition of the endosomal compartments of *Ae. albopictus* midgut cells might provide an advantage for fusion to CHIKV with the E1-A226V mutation [9]. However, the majority of the previously described determinants of vector specificity of different alphaviruses are located within the E2 glycoprotein, circumstantially indicating that the process of alphavirus adaptation to new mosquito species occurs via adaptation to a specific cell surface receptor expressed in this mosquito [27]–[31]. Here, we characterize mutations in the E2 protein that differentially affect *Ae. albopictus* CHIKV midgut infectivity based on the presence of E1-226A or E1-226V residues. Based on our data we conclude that a mutation at position E2-60 influences CHIKV infectivity for *Ae. albopictus*, regardless of the mutations in position E1-226, and also modulates CHIKV infectivity for *Ae. aegypti*. Furthermore, substitutions at E2-211 are crucial for CHIKV sensitivity to the E1-226V mutation in *Ae. albopictus* mosquitoes, but have no effect on CHIKV infectivity for *Ae. aegypti*, and are widely dispersed among CHIKV isolates. These findings provide greater insight into the complexity of the molecular mechanisms involved in adaptation of CHIKV to a new vector.

## Results

### Introduction of the E1-A226V mutation into the backbone of the Ag41855 strain of CHIKV does not lead to a significant increase in infectivity for *Ae. albopictus* mosquitoes

Previously we demonstrated that the introduction of the single amino acid substitution E1-A226V significantly increases CHIKV infectivity for midguts of *Ae. albopictus* mosquitoes [9]. To further investigate the effect of this mutation on infectivity of different strains of CHIKV for *Ae. albopictus*, the E1-A226V mutation was introduced into an enhanced green fluorescent protein (eGFP) expressing infectious clones (i.c.) of the Ag41855 strain of CHIKV (designated as p41855-GFP-226A). The resultant clone was designated p41855-GFP-226V (supplemental data Fig. S1). The specific infectivity values after electroporation of the RNAs produced from p41855-GFP-226A and p41855-GFP-226V were similar - approximately 10^6^ pfu/µg of RNA (supplemental data Table S1), with no detectable differences in plaque sizes. Both constructs provided similar viral titers following *in vitro* transcribed RNA transfection into BHK-21 cells on 1 and 2 days post-electroporation (dpe), indicating that introduction of the E1-A226V mutation into the Ag41855 strain therefore does not attenuate this virus in BHK-21 cells (supplemental data Table S1).

The relative infectivity of 41855-GFP-226V and 41855-GFP-226A viruses in *Ae. albopictus* mosquitoes was determined by oral exposure to serial 10-fold viral dilutions. In two independent experiments the oral infectivities of 41855-GFP-226V and 41855-GFP-E1-226A viruses were not significantly different (p>0.05). This demonstrates that for the Ag41855 strain, the E1-A226V mutation does not affect *Ae. albopictus* midgut infectivity (Table 1). The mean OID_50_ values for 41855-GFP-226V and 41855-GFP-226A were 6.33 and 6.88 Log_10_TCID_50_/ml respectively (Table 1), which was significantly higher compared to OID_50_ values of previously characterized CHIKV strains LR2006 OPY1 and 37997 with either alanine or valine at position E1-226 [9], suggesting that the Ag41855 strain is significantly more attenuated for *Ae. albopictus* infection when compared with the LR2006 OPY1 and 37997 strains.

**Table 1:** Effect of the E1-A226V mutation on infectivity of different strains of CHIKV to *Ae. albopictus* mosquitoes.

Backbone	Exp	E1-226	N m	OID_50_ (C_95_)	OID_50_ mean	p
41855-GFP	1	V	67	6.27 (5.82–6.61)	6.33	p>0.05
	2		54	6.38 (5.94–6.74)		
	1	A	78	6.96 (6.57–7.31)	6.88	
	2		53	6.79 (6.40–7.32)		
LR-GFP	Comb	V	261	NG	3.52	p<0.01
	Comb	A	194	NG	5.45	
37997-GFP	Comb	V	260	NG	3.16	p<0.01
	Comb	A	274	NG	5.10	

Exp – experiment number.

E1-226 – amino acid at position E1-226.

N m – number of mosquitoes used to estimate OID50 value.

OID50 (C95) – oral infectious dose 50 and 95% confidence intervals are expressed as Log10TCID50/ml.

OID50 values and confidence intervals were calculated using PriProbit (Version 1.63).

p – comparison of statistical significance of difference in OID50 values between viruses with E1-226A and E1-226V mutations.

Comb – combined summary of two independent experiments published earlier [9].

NG – value is not given.

The sequence of 41855-GFP-226A was identical to that of the virus stock used for i.c. construction and we previously showed that introduction of the eGFP gene into backbone of LR2006 OPY1 and 37997 strains of CHIKV does not significantly affect infectivity for *Ae. albopictus* and *Ae. aegypti* mosquitoes [32], [33] Tsetsarkin unpublished data]. Nevertheless, we wanted to determine whether or not the attenuation observed for 41855-GFP-226V and 41855-GFP-226A in *Ae. albopictus* could reflect artifacts of the i.c. construction, for example introduction of the eGFP gene under control of an additional sub-genomic promoter or incompatibility of viral segments that were combined in the clone-derived virus but that coexist separately as quasispecies in the viral population. We compared infectivity of the stock Ag41855 virus with that of the viruses produced from the Ag41855 and p41855-GFP-226A clones (Table 2, supplemental data Fig. S1). The OID_50_ values for these viruses were not significantly different (p>0.1) indicating that virus produced from the full-length and eGFP-expressing i.c. retained the infection phenotype of the parental virus in *Ae. albopictus*. This also indicates that the lower infectivity observed for 41855-GFP-226A and 41855-GFP-226V for *Ae. albopictus*, as compared with viruses produced from the i.c. of the LR2006 OPY1 and 37997 strains, is attributed to the specific mutation(s) in the genome of Ag41855 strain.

**Table 2:** Comparison of oral infectivity of the parental virus (Ag41855) and viruses produced from full-length (Ag41855 ic) and eGFP expressing (41855-GFP-226A) i.c. of Ag41855 strain in *Ae. albopictus* mosquitoes.

Virus	N m	OID_50_	C_95_	p
Ag41855	67	6.74	6.39–7.17	p>0.1
Ag41855 ic	78	6.40	6.06–6.85	
41855-GFP-226A	131	6.88^a^	NG	

N m – number of mosquitoes used to estimate OID_50_ value.

OID_50_ and 95% confidence intervals are expressed as Log_10_TCID_50_/ml.

a – average of two independent experiments.

NG – value is not given.

### Sequence comparison of Ag41855, LR2006 OPY1 and 37997 strains

Several recent phylogenetic analyses of CHIKV have grouped the Ag41855 and LR2006 OPY1 strains into the East/Central/South African phylogroup [34], [35] suggesting a close evolutionary relationship. The 37997 strain is a member of the West African phylogroup, which is the outlier among CHIKV strains. Strain 37997 was therefore used as a reference control to identify positions in the Ag41855 genome responsible for attenuation in *Ae. albopictus*, and to determine the sites affecting sensitivity to the E1-A226V mutation in *Ae. albopictus*.

Sequence comparison of the Ag41855 and LR2006 OPY1 strains revealed a total of 202 nucleotide differences (1.7%), encoding 31 amino acid substitutions: 18 in the non-structural and 13 in the structural coding sequence (Table 3). The nucleotide sequence of 37997 differed from Ag41855 and LR2006 OPY1 by 14.7%.

**Table 3:** Genetic difference of Ag41855 and LR2006 OPY1 strains of CHIKV.

	Protein	Ag41855	LR2006 OPY1	37997	Polyprotein
nsP1	326	M	V	M	326^a^
	391	L	F	L	391^a^
	488	Q	R	K	488^a^
nsP2	54	S	N	S	589^a^
	793	A	V	A	1328^a^
nsP3	31	D	G	D	1364^a^
	217	Y	H	Y	1550^a^
	328	**P**	**Q**	**Q**	1661^a^
	337	T	I	T	1670^a^
	358	**P**	**S**	**S**	1691^a^
	435	R	C	H	1768^a^
	438	A	V	V	1771^a^
	449	T	M	A	1782^a^
	461	**L**	**P**	**P**	1794^a^
	471	P	S	P	1804^a^
	524	Stop	R	Stop	1857^a^
nsP4	75	T	A	T	1938^a^
	254	T	A	T	2117^a^
E2	60	**G**	**D**	**D**	385^b^
	162	**V**	**A**	**A**	487^b^
	211	**I**	**T**	**T**	536^b^
	312	T	M	T	637^b^
	318	M	V	T	643^b^
	375	S	T	S	700^b^
	377	V	I	V	702^b^
	386	V	A	V	711^b^
K6	8	V	I	A	756^b^
E1	19	**I**	**V**	**V**	828^b^
	226	A	V	A	1035^b^
	284	D	E	D	1093^b^
	377	**T**	**A**	**A**	1186^b^

a – position in the non-structural polypeptide.

b – position in the structural polypeptide.

Bold type indicates the positions which are the same in LR2006 OPY1 and 37997 strains but different in Ag41855 strain.

A comparison of amino acid sequences for strains Ag41855, LR2006 OPY1 and 37997 (Table 3) identified eight positions that are unique in Ag41855, but are the same in both the LR2006 OPY1 and 37997 strains: three in nsP3 protein (positions 328, 358, and 461), three in the E2 protein (positions 60, 162, and 211) and two in E1 (positions 19 and 377). These data suggest that the unique Ag41855 amino acids could modulate CHIKV infectivity for *Ae. albopictus*. Since numerous previous studies identified the E2 protein as a major determinant of mosquito infectivity for different alphaviruses including Sindbis virus (SINV) and VEEV [27]–[31] we first decided to investigate if the mutations at E2 positions 60, 162, and 211 were responsible for the observed attenuation of the Ag41855 strain, and how these mutations related to the insensitivity of this strain to the E1-A226V mutation in *Ae. albopictus*.

### Determinants of attenuation of Ag41855 strain in *Ae. albopictus* mosquitoes

To elucidate genetic determinants of low mosquito infectivity of strain Ag41855, the fragment of 8021-9225 nt. (which corresponds to 152–553 aa. in the structural polypeptide) from the LR2006 OPY1 i.c. containing the E2 60D, 162A, and 211T mutations, was introduced into 41855-GFP-226V (supplemental data Fig. S1). Based on specific infectivity and replication data, the chimeric virus 41855/LR-GFP-226V was not attenuated in BHK-21 cells (supplemental data Table S1), indicating that this genome region is interchangeable between the LR2006 OPY1 and Ag41855 strains. The 41855/LR-GFP-226V virus was ∼1000 times more infectious for *Ae. albopictus* as compared to Ag41855-GFP-226V virus (OID_50_ = 3.78) (Table 4). The reverse chimera LR/41855-GFP-226V containing the 8021-9225 nt fragment of Ag41855 in the backbone of LR-GFP-226V, demonstrated an OID_50_ value similar to that observed for Ag41855-GFP-226V (OID_50_ = 6.33), indicating that this region encodes the major determinant(s) for *Ae. albopictus* midgut infectivity in the Ag41855 strain. Additionally, these data indicate that mutations in the nsP3 and E1 genes of Ag41855 (Table 3) probably do not affect the *Ae. albopictus* mosquito infectivity phenotype. They therefore were excluded from further analysis.

**Table 4:** Effect of mutations in E2 proteins on CHIKV infectivity for *Ae. albopictus* mosquitoes.

Backbone	Clone name	E1-226	E2-60	E2-162	E2-211	Exp	N m	OID_50_ (C_95_)	OID_50_ mean
41855-GFP	41855-GFP-226V	V	**G**	**V**	**I**	Comb	121	NG	6.33
	NG		*D*	**V**	**I**	1	83	5.51 (5.13–6.13)	5.51
	NG		**G**	*A*	**I**	1	99	6.85 (6.18–9.64)	6.85
	NG		**G**	**V**	*T*	1	125	5.40 (4.94–5.77)	5.40
	NG		**G**	*A*	*T*	1	83	5.57 (5.27–5.83)	5.57
	NG		*D*	**V**	*T*	1	153	3.36 (3.01–3.60)	3.50
						2	105	3.64 (3.31–3.85)	
	NG		*D*	*A*	**I**	1	115	5.52 (5.27–5.80)	5.52
	41855/LR-GFP-226V		*D*	*A*	*T*	1	107	3.78 (2.91–4.08)	3.65
						2	102	3.52 (3.19–3.80)	
	41855-GFP-226A	A	**G**	**V**	**I**	Comb	131	NG	6.88
	NG		*D*	**V**	**I**	1	133	5.69 (5.42–5.92)	5.74
						2	79	5.79 (5.48–6.09)	
	NG		**G**	*A*	**I**	1	75	6.71 (6.44–6.99)	6.71
	NG		**G**	**V**	*T*	1	123	6.51 (6.24–6.78)	6.77
						2	97	7.03 (6.74–7.43)	
	NG		**G**	*A*	*T*	1	98	6.97 (6.68–6.31)	6.97
	NG		*D*	**V**	*T*	1	82	5.48 (5.12–5.79)	5.48
	NG		*D*	*A*	**I**	1	75	5.65 (5.34–5.95)	5.65
	41855/LR-GFP-226A		*D*	*A*	*T*	1	63	5.21 (4.89–5.55)	5.26
						2	134	5.31 (4.96–5.60)	
LR-GFP	LR/41855-GFP-226V	V	**G**	**V**	**I**	1	135	6.40 (5.98–7.28)	6.40
	NG		**G**	*A*	*T*	1	41	5.38 (5.00–5.91)	5.38
	NG		*D*	*A*	**I**	1	120	5.24 (4.92–5.54)	5.24
	NG		*D*	**V**	**I**	1	107	5.52 (5.27–5.80)	5.52
	NG		**G**	*A*	**I**	1	77	6.33 (5.97–6.92)	6.33

Exp – experiment number.

N m – number of mosquitoes used to estimate OID_50_ value.

OID_50_ (C_95_) – oral infectious dose 50 and 95% confidence intervals are expressed as Log_10_TCID_50_/ml.

Comb – combined summary of two independent experiments.

NG – value is not given.

Residues in bold type correspond to authentic amino acids at indicated positions of strain Ag41855. Residues in italics correspond to authentic amino acids at indicated positions of strain LR2006 OPY1.

To further investigate the effect of the 8021–9225 region on CHIKV mosquito infectivity in the background of alanine at E1-226, the 8021-9225 fragment from LR2006 OPY1 was introduced into the 41855-GFP-226A virus (supplemental data Fig. S1). The OID_50_ for the resultant chimeric virus 41855/LR-GFP-226A was 5.21 Log_10_TCID_50_/ml (Table 4) - not significantly different to the OID_50_ values for LR-GFP-226A virus [9]. Importantly, introduction of the 8021–9225 genome fragment of LR2006 OPY1 strain into the background of Ag41855 completely restored the enhancing effect of the E1-A226V mutation on infectivity for *Ae. albopictus* to the levels reported for genetic backgrounds of strains LR2006 OPY1 and 37997 [9].

### Individual role of the mutations at positions E2 60, 162, and 211 on CHIKV infectivity for *Ae. albopictus* mosquitoes

To further characterize the roles of each of the E2 mutations on CHIKV infectivity for *Ae. albopictus*, point mutations encoding amino acids from the LR2006 OPY1 strain were introduced into the backbone of 41855-GFP-226V and 41855-GFP-226A i.c., either individually or in combination (Table 4 and supplemental data Fig. S1). Specific infectivity of *in vitro* transcribed RNA, plaque size and viral titers produced at 1 and 2 dpe were determined for each of the constructs (supplemental data Table S1). All of the constructs were indistinguishable by these parameters, indicating that these mutations do not cause intermolecular incompatibility with the rest of the viral genome and that the resultant viruses are suitable for testing in the *Ae. albopictus*.

Individual introduction of the mutations E2-G60D and E2-I211T into 41855-GFP-226V was responsible for a significant increase of viral infectivity for *Ae. albopictus* to similar levels (OID_50_ values: 5.51 and 5.40 respectively). The OID_50_ of 41855-GFP-226V with the E2-V162A mutation was not significantly different from the OID_50_ for the 41855-GFP-226V virus (Table 4). This suggests that E2-V162A likely plays no role in CHIKV infectivity for *Ae. albopictus*. Interestingly, introduction of each of the three mutations individually did not lead to increase in viral infectivity to the level observed for the triple mutant 41855/LR-GFP-226V, indicating that combinations of at least two mutations are apparently required for the high infectivity phenotype.

Analysis of *Ae. albopictus* midgut infectivity for 41855-GFP-226V, in which two substitutions were introduced into the E2 protein, revealed that a combination of the G60D and I211T mutations is necessary and sufficient to completely restore infectivity of the Ag41855 strain to the same levels as that observed for 41855/LR-GFP-226V and LR-GFP-226V viruses (p>0.1) (Table 4). The *Ae. albopictus* infectivity of viruses where the E2-V162A mutation was combined with either the E2-G60D or E2-I211T mutations, was indistinguishable from the infectivity of the 41855-GFP-226V virus that contained the single mutations in E2-G60D and E2-I211T. This observation further supports the conclusion that position E2-162 does not affect CHIKV infectivity for *Ae. albopictus*. Altogether, these results indicate that there is a strong synergistic effect of the E2-G60D and E2-I211T mutations on CHIKV infectivity for *Ae. albopictus*, when expressed in combination with valine at position E1-226.

To further evaluate the relationships between these different mutations, four additional viruses were constructed in which single and double substitutions at positions E2-60, E2-161 and E2-211 from the Ag41855 strain were substituted into the backbone of LR-GFP-226V (Table 4). The OID_50_ values for LR-GFP-226V with individual mutations E2-D60G and E2-T211I were indistinguishable as compared to OID_50_ values of the 41855-GFP-226V virus with E2-G60D and E2-I211T mutations expressed individually or in combination with E2-A162V, but were significantly higher as compared with LR-GFP-226V. The OID_50_ value for LR-GFP-226V with both the E2-D60G and E2-T211I mutations was indistinguishable from the OID_50_ values of 41855-GFP-226V or chimeric virus LR/41855-GFP-226V (p>0.1). These data suggest that the specific phenotype(s) associated with the E2 mutations introduced into strain Ag41855 would be retained if these mutations were expressed in other CHIKV strains.

The genome region of the LR2006 OPY1 strain that contained mutations at positions E2-60, E2-162 and E2-211 was also responsible for a significant increase in *Ae. albopictus* midgut infectivity for the 41855-GFP-226A virus. In this regard it was important to investigate the individual roles of these particular mutations in the 41855-GFP-226A virus, and to determine how these roles correlated with the effects of these mutations in the 41855-GFP-226V virus. In contrast to the 41855-GFP-226V virus, the introduction of the single mutation E2-G60D into 41855-GFP-226A almost completely restored viral infectivity phenotype to the relatively high levels observed for 41855/LR-GFP-226A and LR-GFP-226A (p>0.1) (Table 4, 1). However, the E2-I211T mutation led to no apparent effect on CHIKV infectivity for *Ae. albopictus*. Analysis of the viruses bearing double mutants in the E2 protein revealed a similar result: viruses containing E2-G60D were significantly more infectious for *Ae. albopictus* than the 41855-GFP-226A virus regardless of the second mutations at positions E2-162 and E2-211. The combination of E2-A162V and E2-I211T did not affect the viral infectivity phenotype as compared to 41855-GFP-226A. In the backbone of 41855-GFP-226A, the E2-G60D and E2-I211T mutations had a disproportionate effect on the CHIKV mosquito infectivity phenotype; where E2-G60D exerted the major effect whilst E2-I211T was responsible for only a marginal effect.

### Ag41855 strain of CHIKV is attenuated in *Ae. aegypti* mosquitoes

Prior to the 2006–2007 outbreaks, *Ae. aegypti* was the principal vector responsible for most urban epidemics of chikungunya [36]. Previously we showed that, in contrast to the situation for *Ae. albopictus* mosquitoes, the E1-A226V mutation does not increase infectivity for *Ae. aegypti* when expressed in the backbones of the LR2006 OPY1 and 37997 strains of CHIKV [9]. Therefore, the unusual phenotype of the Ag41855 strain in *Ae. albopictus* mosquitoes led us to investigate the mosquito infectivity phenotype of this strain in *Ae. aegypti*.

The OID_50_ values of 41855-GFP-226A in *Ae. aegypti* were 6.92 and 7.24 Log_10_TCID_50_/ml, which are significantly higher than the OID_50_ values determined previously for LR-GFP-226A and 37997-GFP-226A viruses (p<0.01) (Table 5). Mosquito infectivity of the 41855-GFP-226V was significantly lower than that of the LR-GFP-226V and 37997-GFP-226V viruses, indicating that the Ag41855 strain of CHIKV is also attenuated in its ability to infect *Ae. aegypti*. Interestingly, 41855-GFP-226A was slightly more infectious for *Ae. aegypti* than 41855-GFP-226V (p_1_<0.05 for the first experiment and p_2_>0.05 for the second). This finding corroborated our previous results showing that CHIKV with the E1-226A residue is slightly more infectious for *Ae. aegypti* mosquitoes [9].

**Table 5:** Effect of the E1-A226V mutation on infectivity of different strains of CHIKV to *Ae. aegypti* mosquitoes.

Backbone	Exp	E1-226	N m	OID_50_ (C_95_)	OID_50_ mean	p
41855-GFP	1	V	112	7.63 (7.35–8.23)	7.71	p_1_<0.05
	2		82	7.78 (7.46–8.21)		p_2_>0.05
	1	A	77	6.92 (6.61–7.24)	7.12	
	2		90	7.24 (6.85–7.66)		
LR-GFP	Comb	V	172	NG	6.52	p_1_<0.05
	Comb	A	156	NG	5.87	p_2_>0.05
37997-GFP	Comb	V	262	NG	6.47	p_1_<0.01
	Comb	A	297	NG	5.70	p_2_>0.05

Exp – experiment number.

E1-226 – amino acid at position E1-226.

N m – number of mosquitoes used to estimate OID_50_ value.

OID_50_ (C_95_) – oral infectious dose 50 and 95% confidence intervals are expressed as Log_10_TCID_50_/ml.

OID_50_ values and confidence intervals were calculated using PriProbit (Version 1.63).

p1 – comparison of statistical significance of difference in OID_50_ values between viruses with E1-226A and E1-226V mutations for experiment 1. p2 – comparison of statistical significance of difference in OID_50_ values between viruses with E1-226A and E1-226V mutations for experiment 2.

Comb – combined summary of two independent experiments published earlier [9].

NG – value is not given.

### Determinants of attenuation of the Ag41855 strain in *Ae. aegypti* mosquitoes

To determine the genomic regions of Ag41855 that are responsible for attenuation of Ag41855 in *Ae. aegypti*, the chimeric viruses 41855/LR-GFP-226A and 41855/LR-GFP-226V as described above, were tested. These viruses were significantly more infectious for *Ae. aegypti* than 41855-GFP-226A and 41855-GFP-226V (p<0.01), with OID_50_ values similar to those of CHIKV possessing either an alanine or a valine at position E1-226 when expressed in the backbone of strains LR2006 OPY1 and 37997 (p>0.1) (Table 6). These data indicate that the 8021–9225 genome region of Ag41855 contains the major determinant of CHIKV attenuation in *Ae. aegypti*. Further analysis of 41855-GFP-226A and 41855-GFP-226V with different combinations of the single or double mutants at positions E2 60, 162, and 211 revealed that introduction of the single mutation E2-G60D was sufficient to increase viral infectivity of the Ag41855 strain for *Ae. aegypti* to the OID_50_ values attributed to CHIKV strains LR2006 OPY1 and 37997 with either alanine or valine at position E1-226 (Table 5, 6). Expression of the E2-60D mutation individually or in combination with E2-A162V or E2-I211T led to a significant decrease in OID_50_ values as compared with the values determined for the 41855-GFP-226A and 41855-GFP-226V viruses (p<0.01). In contrast, introduction of the E2-A162V and E2-I211T mutations individually or in combination resulted in viruses almost indistinguishable from 41855-GFP-226A and 41855-GFP-226V with respect to their ability to infect *Ae. aegypti* midguts, indicating that these two positions do not play important roles in CHIKV transmitted by *Ae. aegypti*.

**Table 6:** OID_50_ for CHIKV in *Ae. aegypti* mosquitoes.

Backbone	Clone name	E1-226	E2-60	E2-162	E2-211	N m	OID_50_	C_95_
41855-GFP	41855-GFP-226V	V	**G**	**V**	**I**	NG	7.71^a^	NG
	NG		*D*	**V**	**I**	105	6.18	5.89–6.64
	NG		**G**	*A*	**I**	61	>7.30	ND
	NG		**G**	**V**	*T*	67	>7.52	ND
	NG		**G**	*A*	*T*	73	>7.31	ND
	NG		*D*	**V**	*T*	52	6.31	5.78–7.14
	NG		*D*	*A*	**I**	81	6.42	6.12–6.85
	41855/LR-GFP-226V		*D*	*A*	*T*	114	6.09	5.81–6.43
	41855-GFP-226A	A	**G**	**V**	**I**	NG	7.12^a^	NG
	NG		*D*	**V**	**I**	83	6.13	5.83–6.46
	NG		**G**	*A*	**I**	103	>7.52	ND
	NG		**G**	**V**	*T*	50	7.30	7.02–7.96
	NG		**G**	*A*	*T*	73	>7.52	ND
	NG		*D*	**V**	*T*	82	6.20	5.97–6.44
	NG		*D*	*A*	**I**	86	6.27	6.04–6.53
	41855/LR-GFP-226A		*D*	*A*	*T*	93	6.23	5.93–6.52

Effect of mutations in E2 proteins on CHIKV infectivity for *Ae. aegypti* mosquitoes.

Exp – experiment number.

N m – number of mosquitoes used to estimate OID_50_ value.

OID_50_ (C_95_) – oral infectious dose 50 and 95% confidence intervals are expressed as Log_10_TCID_50_/ml.

a – average of two independent experiments.

NG – value is not given.

ND – value is not determined.

Residues in bold type correspond to authentic amino acids at indicated positions of strain Ag41855. Residues in italics correspond to authentic amino acids at indicated positions of strain LR2006 OPY1.

### Distribution of the amino acids at E2-60 and E2-211 among characterized CHIKV isolates

The effects of the E2-D60G and E2-T226I on infectivity of CHIKV for *Ae. albopictus* and *Ae. aegypti* that we identified in previous experiments raised an important question regarding the origin of these particular mutations in the genome of the Ag41855 strain, and what evolutionary advantages might be associated with them. To address these questions, we analyzed the distribution of E2-60G and E2-226I mutations among known CHIKV isolates (supplemental data Table S2) and correlated this distribution with their evolution as determined using phylogenetic relationships.

**Figure 1:**
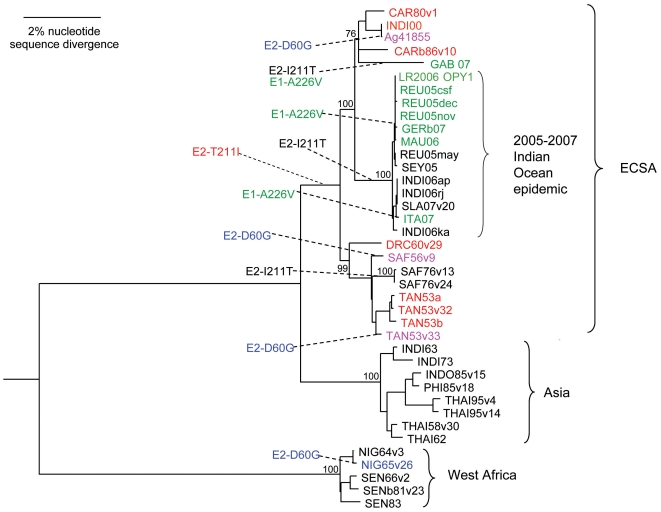
Distribution of the amino acids at E2-60, E2-211 and E1-226 among selected CHIKV isolates. Phylogenetic tree of CHIKV isolates generated using a maximum parsimony algorithm on the 2772 nt. complete E2-K6-E1 genes sequence. Bootstrap analysis was performed with 1000 replicates to determine confidence values on the clades within trees. Character evolution analyses was performed using MacClade4 program. Strains in black – contain E2-60D, E2-211T and E1-226A residues. Strains in red – contain E2-60D, E2-211I and E1-226A residues. Strains in green – contain E2-60D, E2-211T and E1-226V residues. Strains in pink – contain E2-60G, E2-211I and E1-226A residues. Strains in blue – contain E2-60G, E2-211T and E1-226A residues.

The entire genome region encoding the E2-K6-E1 proteins was sequenced or obtained from GenBank and a phylogenetic tree was constructed by the neighbor-joining and maximum parsimony methods followed by bootstrap analysis [37] with 1000 replicates to determine confidence values for the groupings. The phylogeny in Figure 1 reproduces the expected 3 major clades (West African, Asian, and East/Central/South African (ECSA)) [38], with the strains from the recent outbreak evolving as a monophyletic group from the ECSA clade [2]. A manuscript including detailed discussion of the phylogenetic relationship of these strains is currently in preparation (Volk, personal communication).

The E2-60G was present in only four out of 39 CHIKV strains, with no apparent close phylogenetic relationships among them (Fig. 1, supplemental data Table S2). MaClade character evolution analyses indicated that the aspartic acid residue was ancestral, and the glycine residue evolved convergently 4 times. Although no apparent similarities were detected in the passage history of the four strains with the glycine residue (supplemental data Table S2), the presence of different residues in variants of the 1953 Ross strain with different passage histories suggested that the D60G substitution may have been selected by cell culture or animal passage.

In contrast to E2-60G, the E2-211I had a very different distribution among CHIKV isolates (Fig. 1). It was present in 10 of 39 strains, all belonging to the ECSA clade. Interestingly, this residue was found to be even more prevalent in this phylogroup among strains isolated before 2005 as compared to E2-211T. These data suggest that the E2-211I might be important for adaptation of CHIKV to some particular conditions prevalent in these regions, for example specific vectors or vertebrates involved in the sylvatic transmission cycle. Within the ECSA clade, E2-211T was found in all isolates from the 2005–2007 CHIKV outbreak and in two more strains isolated in 1976 from the South African Republic (Fig. 1, supplemental data Table S2). Character analyses indicated that the E2-I211T substitution probably occurred convergently on three separate occasions within the ECSA clade, leading to South African 1976 strains, Indian Ocean 2005–2007 strains and Gabonese 2007 strain (Fig. 1). This conclusion is supported by the presence of the E2-211I residue in two strains from Comoros isolated in 2005 [39] belonging to the Indian Ocean clade. The sequences for these strains are unavailable in GenBank which precluded us from including them in our phylogenetic analysis. Finally, as determined previously [2], [5], our analyses indicated at least three convergent E1-A226V replacements during the recent epidemics in the Indian Ocean and India (Fig. 1).

## Discussion

In this work we performed a detailed investigation of the genetic factors responsible for the relatively low infectivity of the Ag41855 strain for *Ae. albopictus* and *Ae. aegypti* mosquitoes. Comprehensive analyses identified amino acid residues at E2-60 and E2-211 that modulate the role of the E1-A226V mutation that first arose during the Indian Ocean epidemic [2], [5] on CHIKV infectivity in these vectors. Individual expression of the E2-G60D or E2-I211T mutation in the 41855-GFP-226V virus has an identical effect on the OID_50_ for *Ae. albopictus*, and their combined expression increases infectivity of strain Ag41855, into which the E1-A226V mutation was introduced, to the level characteristic for strains LR2006 OPY1 and 37997. However, expression of only E2-G60D (but not E2-I211T) was necessary and sufficient to elevate the *Ae. albopictus* infectivity of the Ag41855 strain with an alanine at E1-226. When considered together, these data provide new and important insights into the roles played by E2-G60D and E2-211 mutations in determining CHIKV infectivity for *Ae. albopictus* mosquitoes.

The mutation E2-G60D significantly increases Ag41855 infectivity for *Ae. albopictus* when expressed with either alanine or valine at E1-226. This indicates that residue E2-60D is important for vector infectivity of CHIKV, but it does not specifically affect the previously observed phenotypes reported for the E1-A226V mutation. This conclusion was further supported by the results obtained from *Ae. aegypti* infectivity experiments. In this mosquito, the E1-A226V mutation does not increase CHIKV infectivity [9] and therefore this vector can be used as a species-specificity control. In *Ae. aegypti*, expression of the E2-60D alone was necessary and sufficient to increase Ag41855 infectivity to that of the LR2006 OPY1 and 37997 levels. Interestingly, the E2-60G residue was found only in three other CHIKV strains, none of which share close phylogenetic relationships. More importantly, the IND-00 CHIKV strain, which is almost 100% identical to the Ag41855 strain based on both nucleotide and amino acid sequences, has a glutamic acid residue at E2-60. This suggests that acquisition of E2-D60G occurs sporadically, possibly during propagation of the virus under laboratory conditions. Altogether, accumulated data suggests that the E2-60 residue affects some basic mechanism used by CHIKV to infect its vector. Disruption of this mechanism exerts a strong inhibitory effect on CHIKV infectivity to both *Ae. albopictus* and *Ae. aegypti*, and therefore this mutation should be quickly eliminated from naturally transmitted strains.

To our knowledge, this is the first report incriminating alphavirus position E2-60 as a major determinant of mosquito infectivity. Interestingly, a recent study demonstrated that introduction of two mutations; E2-H55Q and E2-K70E from the TR339 strain into the TE/5'2J of SINV increased infectivity for *Ae. aegypti*, both independently and in combination [30]. These mutants are located in close proximity to, and share similar properties with E2-G60D; they both decrease the net positive charge of the E2 protein, indicating that their mechanism of action in SINV and CHIKV may be the same. Currently, the particular role of mutations that increase the charge in this region of the E2 protein is uncertain. Previous studies demonstrated that the E2-Q55H substitution leads to increased SINV neurovirulence in mice [40], and also increases binding to neuroblastoma cells. However, E2-E70K was shown to reduce neurovirulence in neonatal mice [41]. It would be interesting to investigate possible association between mutations at E2-60 and the rare neurological complications and fatalities that were reported for the first time during the recent chikungunya epidemics [42]. Both mutations E2-Q55H and E2-E70K were also found to be involved in increased SINV binding to heparan sulfate (HS) [40], [43] which is in agreement with the hypothesis that the E2-D60G substitution occurred in the Ag41855 strain as a result of adaptation of the virus to sulfated proteoglycans abundantly expressed on the cell surface of BHK-21 and Vero cells. Although, to our knowledge there are no examples of HS adaptation due to mutations which lead to loss of a negative charge in E2 protein for any alphaviruses, it has been shown that E to G mutation at position 122 of the E protein of Tick-Borne Encephalitis Virus (TBEV) (a member of family *Flaviviridae*) mediates adaptation of TBEV to BHK-21 cells via increasing virus binding to HS [44].

In contrast to the E2-G60D, the mutation E2-I211T significantly increases infectivity of the Ag41855 strain for *Ae. albopictus* only when expressed together with the E1-226V mutation. If CHIKV has the pre-2005 E1-226A, then the substitution E2-211T has almost no effect on infectivity for *Ae. albopictus* compared with the E2-211I variant. This indicates that the E2-I211T mutation is responsible for specific modulation of CHIKV infectivity for *Ae. albopictus*. The results of CHIKV infectivity for *Ae. aegypti* further support this conclusion. In *Ae. aegypti*, the E1-A226V mutation does not increase CHIKV infectivity [9] and in the current study, the E2-I211T mutation did not affect infectivity for these mosquitoes. Altogether, these mosquito infectivity data indicate that the E1-A226V would not give a selective advantage to the CHIKV strains possessing E2-211I with respect to transmissibility by *Ae. albopictus*. Such viruses would probably not be selected in nature. This conclusion is in agreement with our phylogenetic analyses. It has been shown that the E1-A226V mutation appeared independently at least three times in strains of CHIKV transmitted by *Ae. albopictus* [5], but it is important to note that all of these CHIKV isolates had threonine at position E2-211.

Phylogenetic analysis of the distribution of the E2-211I mutation revealed substantial differences compared to the E2-60G mutation. E2-211I was present in the majority of pre-2005 CHIKV isolates in the ECSA complex, suggesting that it might play an important role for maintenance of CHIKV in the enzootic African cycle involving wild non-human primates and forest-dwelling *Aedes* spp. mosquitoes. Based on isolation frequencies, the main sylvatic vectors of CHIKV are probably *Ae. furcifer-taylori*, *Ae. africanus* and *Ae. luteocephalus* [45], with *Ae. furcifer-taylori* more important in southern and western Africa [46], [47] and *Ae. africanus* more important in central regions [45], [48], [49]. Since the E2-211I mutation was predominantly found in the CHIKV strains isolated in central Africa, it is possible that this mutation might give a selective advantage to CHIKV transmitted by *Ae. africanus*. The involvement of the particular species of non-human primates as vertebrate hosts for CHIKV in central Africa is yet to be analyzed.

We cannot exclude the possibility that the observed predominance of E2-211I among CHIKV strains within the ECSA complex arose not due to some selective advantage but rather due to a founder effect. It is possible that the ancestral progenitor of these viruses had an isoleucine at E2-211 and the absence of the selective pressure at this position led to fixation of this mutation in the viral population. It is also possible that CHIKV strains with T and I at E2-211 coexist in nature, and changes at this position occur due to some, as of yet unidentified, conditions. In this case, the apparent prevalence of E2-211I among ECSA strains isolated before 2005 could be an artifact resulting from the limited numbers of CHIKV strains available for analysis. This hypothesis is supported by recent studies of CHIKV evolution on Comoros and Reunion islands [2], [39]. It was shown that the strains of CHIKV which caused the 2005–2006 outbreak on Reunion were almost identical to those isolated during the 2005 outbreak on Comoros island. Two out of three sequenced isolates from Comoros have E2-211I and one has E2-211T, indicating that both variants were simultaneously transmitted in the region [39]. Interestingly, *Ae. aegypti* mosquitoes were the main vector in this initial epidemic [39], which is consistent with our observation that the E2-I211T mutation does not affect CHIKV infectivity for this vector. The precise distribution of E2-211I and E2-211T residues among early strains (prior to the appearance of the E1-A226V mutation) from the 2005–2006 outbreak on Reunion is unknown. However, the presence of only E2-211T in three of three sequences with the E1-226A [2] circumstantially indicates that E2-211T was probably the predominant variant on Reunion Island.

Position E2-211 is located within, or in close proximity to, the sites that have previously been shown to harbor genetic determinants of host specificity for several alphaviruses. Substitution E2-S218N was responsible for increased VEEV subtype IE infectivity to *Ochlerotatus (Aedes) taeniorhynchus* mosquitoes [29]. The deletion of E2-200-220 in SINV significantly decreases infectivity of the strain MRE16 to *Ae. aegypti* [27]. Mutations at E2-T213R of VEEV [50] and E2-T219A of Ross River virus (RRV) [51] were also shown to be responsible for adaptation of these viruses to a new host species. It was suggested that this region of the E2 protein constitutes a cell-receptor binding domain [11] and mutations here might affect mosquito infectivity by disruption of the proper interactions of alphavirus with their receptor(s) expressed on midgut cells [27]. The atomic structure of E2 has not been solved for any alphavirus, but several lines of evidence indicate that the regions around position E2-211 are exposed on the virion surface and are involved in interactions with cellular receptors [11]. Analysis of SINV escape mutants resistant to six neutralizing monoclonal antibodies (MAb) identified that all changes occurred between residues 183-216 of the E2 protein, suggesting that this region constitutes prominent antigenic domain(s) that interact directly with neutralizing antibodies [52]. In an alternative approach using λgt11 clones expressing parts of E2, the same 183–216 aa. region was found to interact with five MAbs reactive to E2 protein [53]. Anti-idiotypic antibodies produced to one of the MAbs (MAb49) which was used by [52], [53] blocked SINV binding by up to 50% and were capable of immunoprecipitating a 63 kD protein from chicken cell's plasma membranes. Interestingly the SINV escape mutant for MAb49 has a single aa. substitution E2-R214P [54]. Cryoelectron microscopy of RRV complexed with the Fab fragment of MAb T10C9, which binds in the vicinity of the E2-216 residue, revealed that the binding region is located at the outermost tip of the E2 glycoprotein [55]. Cryoelectron microscopy followed by image reconstruction of HS-adapted RRV revealed that HS binds in the same outermost region of the E2 glycoprotein as MAb T10C9 [56]. The E2-N218K mutation, which was responsible for adaptation of RRV to HS binding, was also responsible for resistance of the virus to neutralization by MAb T10C9 [57]. Interestingly the E2-N218K mutation was originally selected for replication of RRV in chicken embryo fibroblast cells and was shown to attenuate the virus in 1-day old mice [58]. This demonstrates that changes in this region of the E2 glycoprotein expand the host range of RRV in cell culture by allowing virus to interact with cell surface HS moieties.

Considering the evidence for the involvement of mutations in the region around position E2-211 in receptor binding and in adaptation to new host species, we believe that the simplest explanation for the specific effects of the E2-211I mutation on CHIKV infectivity for *Ae. albopictus* mosquitoes is that this substitution disrupts the ability of CHIKV to interact with a particular receptor on the midgut epithelial cells of *Ae. albopictus*. This receptor might be responsible for targeting of CHIKV to the specific endosomal compartments/domains with a unique lipid composition that favors fusion of CHIKV possessing valine at E1-226, as compared to alanine. If this pathway is blocked by the E2-211I mutation then CHIKV may infect *Ae. albopictus* mosquitoes using an alternative receptor(s) that targets virus into different endosomes, in which the presence of the E1-A226V does not result in differential infectivity. It is also tempting to suggest that, in *Ae. aegypti* mosquitoes, only the second (alternative) pathway is available for CHIKV infection, thereby making this species insensitive to the E1-A226V mutation. In agreement with this hypothesis is the fact that mutations at position E1-226 could be responsible for modulation of the lipid requirement for growth of CHIKV [9], Semliki Forest virus [59] and SINV [60] in C6/36 cells.

Alternatively, mutations at E2-211 might affect the stability of the E2-E1 heterodimers, which would differentially affect fusion properties of the CHIKV with E1-226A or E1-226V. It has been shown that the mutation E2-D216G can rescue a PE2 cleavage mutant of SINV by disrupting the E2-E1 heterodimer stability under acidic condition [61]. However, there were no significant differences in the pH threshold for membrane fusion or cholesterol dependence of CHIKV containing E2-211T or E2-211I (data not shown). These data indicate that that these mutations probably affect steps in CHIKV cells entry preceding fusion.

Additional studies are required to investigate the precise molecular mechanisms responsible for the observed, unique roles of the substitutions at position E2-60 and E2-211 of CHIKV. The widespread and increasing distribution of *Ae. albopictus* [13], [14] represents a potential threat with respect to the spread and establishment of CHIKV in other tropical and temperate regions. The current study builds upon our previous work and reveals that mosquito species-specificity of CHIKV, and potentially of other important human and animal pathogens, for example VEEV, can be influenced by multiple genes that can act synergistically. Understanding the complex virus-vector interactions and their underlying mechanisms is critical to enhance our capacity to assess the risks of epidemic emergence. Furthermore, understanding these interactions may also reveal targets that can be exploited for the design of antiviral strategies to modify viral infectivity/attenuation and identify cellular molecules and pathways involved in the infection process.

## Materials and Methods

### Viruses and plasmids

The plasmid encoding eGFP-expressing full-length cDNA clone derived from CHIKV LR2006 OPY1 strain LR-GFP-226V (CHIK-LR 5'GFP, accession number EU224269) has been previously described [9], [32]. The plasmids encoding full-length and full-length eGFP-expressing i.c. of the Ag41855 strain of CHIKV were generated using methodology similar to those described previously for CHIKV strains LR2006 OPY1 and 37997 [32], [33]. The Ag41855 strain of CHIKV was obtained from the World Reference Center for Emerging Viruses and Arboviruses at the University of Texas Medical Branch, Galveston, TX. This strain was isolated in 1982 in Uganda from a human and was passed tree times in suckling mice and twice in Vero cells before being used for i.c. construction. The Ag41855 strain of CHIKV was chosen because of its close phylogenetic relationship to the strains implicated in the 2006–2007 epidemics, and therefore represented an interesting model for studying the evolutionary events that preceded these epidemics.

All plasmids were constructed and propagated using conventional cloning methods [62]. The integrity of PCR-generated cDNAs was verified by sequence analysis. All plasmids were purified by either centrifugation in cesium chloride gradients or by using QIAGEN Plasmid Mini Kits (QIAGEN, Valencia, CA) following the manufacturer's instructions. Plasmid maps, sequences and detailed descriptions of all constructs are available from the authors upon request.

All plasmids were linearized with *Not*I and *in vitro* transcribed from the minimal SP6 promoter using the mMESSAGE mMACHINE kit (Ambion, Austin, Texas) following the manufacturer's instructions. The yield and integrity of synthesized RNA were analyzed by agarose gel electrophoresis in the presence of 0.25 µg/ml of ethidium bromide. RNA (∼10 µg) was electroporated into 1×10^7^ BHK-21 (baby hamster kidney) cells as previously described and cells were then transferred to 75 cm^2^ tissue culture flasks with 15 ml of Leibovitz L-15 (L-15) medium. Supernatants were collected at 24 and 48 h post-electroporation and stored at −80°C. Electroporation efficiency was estimated using an infectious centers assay as previously described [32]. Briefly, 1×10^5^ electroporated BHK-21 cells were serially 10-fold diluted and seeded in six-well plates containing 10^6^ Vero (green monkey kidney) cells per well in MEM media. Following an incubation for 2 h at 37°C, cells were overlaid with 2 mL of 0.5% agarose containing MEM supplemented with 3.3% FBS. Cells were incubated for 2 d at 37°C until plaques developed and were stained with crystal violet.

### Cells and mosquitoes

BHK-21 cells were maintained at 37°C in L-15 medium supplemented with 10% fetal bovine serum (FBS), 100 U penicillin, and 100 µg/ml streptomycin. *Ae. aegypti* (white-eyed Higgs variant of the Rexville D strain) and *Ae. albopictus* (Galveston strain) were reared at 27°C and 80% relative humidity under a 16 h light: 8 h dark photoperiod, as previously described [33]. Adults were kept in paper cartons supplied with 10% sucrose on cotton balls. To promote egg production, females were fed on anaesthetized hamsters once per week. All animal manipulations were performed in accordance with National Institutes of Health and University of Texas Medical Branch Institutional Animal Care and Use Committee (http://research.utmb.edu/iacuc/) approved protocols.

Viral titers from tissue culture supernatants were determined by titration on Vero cells and expressed as tissue culture infectious dose 50 percent endpoint titers (Log_10_TCID_50_/ml) as previously described [63].

### Oral infection of mosquitoes

Most of the studies of oral infectivity of CHIKV in *Ae. aegypti* and *Ae. albopictus* mosquitoes were performed using eGFP-expressing viruses. *Ae. aegypti* and *Ae. albopictus* were infected in an Arthropod Containment Level 3 insectary as described previously [64], [65]. To estimate the Oral Infectious Dose 50% values (OID_50_), frozen stocks of viruses were thawed at 37°C and four to five 10-fold serial dilutions of virus were made in L-15 medium followed by mixing the samples with an equal volume of defibrinated sheep blood. Each viral dilution was presented to 50 4–5-day post-eclosion *Ae. aegypti* or *Ae. albopictus* female mosquitoes (starved for 24 h) using a Hemotek membrane feeding system (Discovery Workshops, Accrington, Lancashire, United Kingdom) fitted with a murine skin membrane. Mosquitoes were permitted to feed for 1 h, after which engorged mosquitoes [stage>3b [66]] returned to the cages for maintenance.

At 7 days post-infection (dpi) mosquitoes were dissected and eGFP expression in infected midguts was analyzed by fluorescence microscopy. A mosquito was considered infected if at least one focus of eGFP-expressing cells was present in the midgut. To compare oral infectivity of non-eGFP-expressing viruses 16 to 24 mosquitoes from each viral dilution were collected on day 7 post-infection, individually triturated in 1 ml of L-15 media and titrated as described [33]. A mosquito was considered infected if it contained more than 0.94 Log_10_TCID_50_ infection units (limit of detection). The experiments were performed once or twice for each virus. OID_50_ values and confidence intervals were calculated using PriProbit program (version 1.63). The SAS equivalent method was used to calculate the fiducial limits (confidence intervals), assuming normal function distribution and an “all or nothing” response parameter. The difference between two OID_50_ values was considered statistically significant if 95% fiducial limits did not overlap.

### Phylogenetic analyses

Entire E2-K6-E1 genome region of the selected CHIKV strains were sequenced or obtained from GenBank. Viruses whose genomes were not available in GenBank were obtained from the World Reference Center for Emerging Viruses and Arboviruses at the University of Texas Medical Branch, Galveston, TX. Viruses were passaged in C6/36 *Ae. albopictus* cells, concentrated using polyethylene glycol (7% W/V) and NaCl (2.3% (W/V), and RNA was extracted using TRIzol (Invitrogen, Carlsbad, CA) according to the manufacturer's protocol. Overlapping PCR amplicons were amplified from viral RNA using the Titan One Tube RT-PCR system (Roche, Mannheim, Germany) according to the manufacturer's protocol. Primer sequences and specific PCR protocols are available from the authors upon request. Agarose gel-purified amplicons were sequenced directly using BigDye Terminator v3.1 cycle sequencing kit (Applied Biosystems, Foster City, CA; primer sequences available upon request) designed from a genomic alignment of strains 37997, LR2006 OPY1, 15561, TSI-GSD-218, Ross, and RSU1 sequence. Sequencing was performed in an ABI PRISM model 3100 Genetic Analyzer (Applied Biosystems, Foster City, CA), and sequences were edited and assembled in VectorNTI v10.3 (Invitrogen, www.invitrogen.com). Newly-generated CHIKV sequences as well as those available from the GenBank library, along with that of o'nyong-nyong virus (ONNV, strain Gulu, used as an outgroup) were aligned with ClustalW in MacVector v9.0 (MacVector, Inc., Cary, NC) or BioEdit v7.0.5.3 (http://www.mbio.ncsu.edu/BioEdit/bioedit.html); gap introductions in the nucleotide alignment were refined using amino acid alignments to preserve codon homology. The phylogeny was produced using neighbor-joining and maximum parsimony methods available in the PAUP* v4.0b 10 package (Sinauer Associates, Inc., Sunderland, MA). Bootstrap analysis [37] was performed with 1000 replicates to assess reliability of the grouping.

## Supporting Information

Figure S1 Schematic representation of the viruses used in this study.
(1.20 MB TIF)

Table S1 Recovery of the viruses with mutations in E2 protein after electroporation of in vitro transcribed RNA. a - amino acids at position of E1-226. b - amino acids at position of E2: 60, 162, 211. c - Specific infectivity of in vitro transcribed RNA. 10(7) BHK-21 cells were transfected with 10 µg of RNA. Electroporated BHK-21 cells were ten fold serially diluted, seeded in 6 well tissue culture plates containing 1×10(6) Vero cells per well in MEM media. Following an incubation for 2 h at 37°C, cells were overlaid with 2 mL of 0.5% agarose containing 3.3% FBS in MEM. Plaques were scored and measured on day 2 post transfection. d - Supernatants of electroporated BHK-21 cells were collected on days 1 and 2. Virus titers were determined by titration on Vero cells and expressed as Log10TCID50/ml. e - Plaque size of infectious centers expressed in millimeters±standard deviation. h - hours post-infection. Blue color corresponds to authentic amino acids at indicated positions of strain Ag41855, red color corresponds to authentic amino acids at indicated positions of strain LR2006 OPY1.
(0.06 MB DOC)

Table S2 Summary of virus strains used in phylogenetic analysis. Genotype: ECSA - Eastern/Central/South African; W. Afr - West African. Passage history: SM: suckling mouse; C6/36: Ae. albopictus cell line; Vero: African green monkey cell line; RMK: Rhesus monkey kidney cell line; MRC-5: human lung epithelium; AP61: Ae. pseudoscutellaris cell line. GenBank Acc - GenBank accession number. ? - information is unavailable to the authors
(0.10 MB DOC)

## References

[1] Simon F, Savini H, Parola P (2008). Chikungunya: a paradigm of emergence and globalization of vector-borne diseases.. Med Clin North Am.

[2] Schuffenecker I, Iteman I, Michault A, Murri S, Frangeul L (2006). Genome microevolution of chikungunya viruses causing the Indian Ocean outbreak.. PLoS Med.

[3] Reiter P, Fontenille D, Paupy C (2006). *Aedes albopictus* as an epidemic vector of chikungunya virus: another emerging problem?. Lancet Infect Dis.

[4] Delatte H, Paupy C, Dehecq JS, Thiria J, Failloux AB (2008). [*Aedes albopictus*, vector of chikungunya and dengue viruses in Reunion Island: biology and control].. Parasite.

[5] de Lamballerie X, Leroy E, Charrel RN, Ttsetsarkin K, Higgs S (2008). Chikungunya virus adapts to tiger mosquito via evolutionary convergence: a sign of things to come?. Virol J.

[6] Bonilauri P, Bellini R, Calzolari M, Angelini R, Venturi L (2008). Chikungunya virus in *Aedes albopictus*, Italy.. Emerg Infect Dis.

[7] Enserink M (2008). Entomology. A mosquito goes global.. Science.

[8] Pages F, Peyrefitte CN, Mve MT, Jarjaval F, Brisse S (2009). *Aedes albopictus* mosquito: the main vector of the 2007 Chikungunya outbreak in Gabon.. PLoS ONE.

[9] Tsetsarkin KA, Vanlandingham DL, McGee CE, Higgs S (2007). A Single Mutation in Chikungunya Virus Affects Vector Specificity and Epidemic Potential.. PLoS Pathog.

[10] Vazeille M, Moutailler S, Coudrier D, Rousseaux C, Khun H (2007). Two Chikungunya Isolates from the Outbreak of La Reunion (Indian Ocean) Exhibit Different Patterns of Infection in the Mosquito, *Aedes albopictus*.. PLoS ONE.

[11] Strauss JH, Strauss EG (1994). The alphaviruses: gene expression, replication, and evolution.. Microbiol Rev.

[12] Kielian M, Rey FA (2006). Virus membrane-fusion proteins: more than one way to make a hairpin.. Nat Rev Microbiol.

[13] Gratz NG (2004). Critical review of the vector status of *Aedes albopictus*.. Med Vet Entomol.

[14] Benedict MQ, Levine RS, Hawley WA, Lounibos LP (2007). Spread of the tiger: global risk of invasion by the mosquito *Aedes albopictus*.. Vector Borne Zoonotic Dis.

[15] Rezza G, Nicoletti L, Angelini R, Romi R, Finarelli AC (2007). Infection with chikungunya virus in Italy: an outbreak in a temperate region.. Lancet.

[16] Enserink M (2007). EPIDEMIOLOGY: Tropical Disease Follows Mosquitoes to Europe.. Science.

[17] Mitchell CJ, Niebylski ML, Smith GC, Karabatsos N, Martin D (1992). Isolation of eastern equine encephalitis virus from *Aedes albopictus* in Florida.. Science.

[18] Scott TW, Lorenz LH, Weaver SC (1990). Susceptibility of *Aedes albopictus* to infection with eastern equine encephalomyelitis virus.. J Am Mosq Control Assoc.

[19] Fernandez Z, Moncayo AC, Carrara AS, Forattini OP, Weaver SC (2003). Vector competence of rural and urban strains of *Aedes* (Stegomyia) *albopictus* (Diptera: Culicidae) from Sao Paulo State, Brazil for IC, ID, and IF subtypes of Venezuelan equine encephalitis virus.. J Med Entomol.

[20] Beaman JR, Turell MJ (1991). Transmission of Venezuelan equine encephalomyelitis virus by strains of *Aedes albopictus* (Diptera: Culicidae) collected in North and South America.. J Med Entomol.

[21] Mitchell CJ, Miller BR, Gubler DJ (1987). Vector competence of *Aedes albopictus* from Houston, Texas, for dengue serotypes 1 to 4, yellow fever and Ross River viruses.. J Am Mosq Control Assoc.

[22] Sardelis MR, Turell MJ, O'Guinn ML, Andre RG, Roberts DR (2002). Vector competence of three North American strains of *Aedes albopictus* for West Nile virus.. J Am Mosq Control Assoc.

[23] Sardelis MR, Turell MJ, Dohm DJ, O'Guinn ML (2001). Vector competence of selected North American *Culex* and *Coquillettidia* mosquitoes for West Nile virus.. Emerg Infect Dis.

[24] Turell MJ, O'Guinn ML, Dohm DJ, Jones JW (2001). Vector competence of North American mosquitoes (Diptera: Culicidae) for West Nile virus.. J Med Entomol.

[25] Weng MH, Lien JC, Wang YM, Wu HL, Chin C (1997). Susceptibility of three laboratory strains of *Aedes albopictus* (Diptera: Culicidae) to Japanese encephalitis virus from Taiwan.. J Med Entomol.

[26] Moutailler S, Krida G, Schaffner F, Vazeille M, Failloux AB (2008). Potential vectors of Rift Valley fever virus in the Mediterranean region.. Vector Borne Zoonotic Dis.

[27] Myles KM, Pierro DJ, Olson KE (2003). Deletions in the putative cell receptor-binding domain of Sindbis virus strain MRE16 E2 glycoprotein reduce midgut infectivity in *Aedes aegypti*.. J Virol.

[28] Brault AC, Powers AM, Weaver SC (2002). Vector infection determinants of Venezuelan equine encephalitis virus reside within the E2 envelope glycoprotein.. J Virol.

[29] Brault AC, Powers AM, Ortiz D, Estrada-Franco JG, Navarro-Lopez R (2004). Venezuelan equine encephalitis emergence: enhanced vector infection from a single amino acid substitution in the envelope glycoprotein.. Proc Natl Acad Sci U S A.

[30] Pierro DJ, Powers EL, Olson KE (2007). Genetic determinants of Sindbis virus strain TR339 affecting midgut infection in the mosquito *Aedes aegypti*.. J Gen Virol.

[31] Pierro DJ, Powers EL, Olson KE (2008). Genetic determinants of Sindbis virus mosquito infection are associated with a highly conserved alphavirus and flavivirus envelope sequence.. J Virol.

[32] Tsetsarkin K, Higgs S, McGee CE, De Lamballerie X, Charrel RN (2006). Infectious clones of Chikungunya virus (La Reunion isolate) for vector competence studies.. Vector Borne Zoonotic Dis.

[33] Vanlandingham DL, Tsetsarkin K, Hong C, Klingler K, McElroy KL (2005). Development and characterization of a double subgenomic chikungunya virus infectious clone to express heterologous genes in *Aedes aegypti* mosquitoes.. Insect Biochem Mol Biol.

[34] Arankalle VA, Shrivastava S, Cherian S, Gunjikar RS, Walimbe AM (2007). Genetic divergence of Chikungunya viruses in India (1963–2006) with special reference to the 2005–2006 explosive epidemic.. J Gen Virol.

[35] Cherian SS, Walimbe AM, Jadhav SM, Gandhe SS, Hundekar SL (2009). Evolutionary rates and timescale comparison of Chikungunya viruses inferred from the whole genome/E1 gene with special reference to the 2005–07 outbreak in the Indian subcontinent.. Infect Genet Evol.

[36] Jupp PG, McIntosh BM, P. MT (1988). Chikungunya virus disease.. The Arboviruses: Epidemiology and Ecology.

[37] Felsenstein J (1985). Confidence limits on phylogenies: an approachusing the bootstrap.. Evolution.

[38] Powers AM, Brault AC, Tesh RB, Weaver SC (2000). Re-emergence of Chikungunya and O'nyong-nyong viruses: evidence for distinct geographical lineages and distant evolutionary relationships.. J Gen Virol.

[39] Kariuki Njenga M, Nderitu L, Ledermann JP, Ndirangu A, Logue CH (2008). Tracking epidemic Chikungunya virus into the Indian Ocean from East Africa.. J Gen Virol.

[40] Levine B, Griffin DE (1993). Molecular analysis of neurovirulent strains of Sindbis virus that evolve during persistent infection of scid mice.. J Virol.

[41] McKnight KL, Simpson DA, Lin SC, Knott TA, Polo JM (1996). Deduced consensus sequence of Sindbis virus strain AR339: mutations contained in laboratory strains which affect cell culture and in vivo phenotypes.. J Virol.

[42] Arpino C, Curatolo P, Rezza G (2009). Chikungunya and the nervous system: what we do and do not know.. Rev Med Virol.

[43] Smit JM, Waarts BL, Kimata K, Klimstra WB, Bittman R (2002). Adaptation of alphaviruses to heparan sulfate: interaction of Sindbis and Semliki forest viruses with liposomes containing lipid-conjugated heparin.. J Virol.

[44] Mandl CW, Kroschewski H, Allison SL, Kofler R, Holzmann H (2001). Adaptation of tick-borne encephalitis virus to BHK-21 cells results in the formation of multiple heparan sulfate binding sites in the envelope protein and attenuation in vivo.. J Virol.

[45] Jupp PG, McIntosh BM, Monath TP (1988). Chikungunya virus disease.. The Arboviruses: Epidemiology and Ecology.

[46] McIntosh BM, Jupp PG, Dos Santos I (1977). Rural epidemic of chikungunya in South Africa with involvement of *Aedes (Diceromyia) furcifer* (Edwards) and baboons.. South African Journal of Science.

[47] Diallo M, Thonnon J, Traore-Lamizana M, Fontenille D (1999). Vectors of Chikungunya virus in Senegal: current data and transmission cycles.. Am J Trop Med Hyg.

[48] Weinbren MP, Haddow AJ, Williams MC (1958). The occurrence of Chikungunya virus in Uganda. I. Isolation from mosquitoes.. Trans R Soc Trop Med Hyg.

[49] McCrae AW, Henderson BE, Kirya BG, Sempala SD (1971). Chikungunya virus in the Entebbe area of Uganda: isolations and epidemiology.. Trans R Soc Trop Med Hyg.

[50] Anishchenko M, Bowen RA, Paessler S, Austgen L, Greene IP (2006). Venezuelan encephalitis emergence mediated by a phylogenetically predicted viral mutation.. Proc Natl Acad Sci U S A.

[51] Burness AT, Pardoe I, Faragher SG, Vrati S, Dalgarno L (1988). Genetic stability of Ross River virus during epidemic spread in nonimmune humans.. Virology.

[52] Strauss EG, Stec DS, Schmaljohn AL, Strauss JH (1991). Identification of antigenically important domains in the glycoproteins of Sindbis virus by analysis of antibody escape variants.. J Virol.

[53] Wang KS, Strauss JH (1991). Use of a lambda gt11 expression library to localize a neutralizing antibody-binding site in glycoprotein E2 of Sindbis virus.. J Virol.

[54] Wang KS, Schmaljohn AL, Kuhn RJ, Strauss JH (1991). Antiidiotypic antibodies as probes for the Sindbis virus receptor.. Virology.

[55] Smith TJ, Cheng RH, Olson NH, Peterson P, Chase E (1995). Putative receptor binding sites on alphaviruses as visualized by cryoelectron microscopy.. Proc Natl Acad Sci U S A.

[56] Zhang W, Heil M, Kuhn RJ, Baker TS (2005). Heparin binding sites on Ross River virus revealed by electron cryo-microscopy.. Virology.

[57] Heil ML, Albee A, Strauss JH, Kuhn RJ (2001). An amino acid substitution in the coding region of the E2 glycoprotein adapts Ross River virus to utilize heparan sulfate as an attachment moiety.. J Virol.

[58] Kerr PJ, Weir RC, Dalgarno L (1993). Ross River virus variants selected during passage in chick embryo fibroblasts: serological, genetic, and biological changes.. Virology.

[59] Vashishtha M, Phalen T, Marquardt MT, Ryu JS, Ng AC (1998). A single point mutation controls the cholesterol dependence of Semliki Forest virus entry and exit.. J Cell Biol.

[60] Lu YE, Cassese T, Kielian M (1999). The cholesterol requirement for sindbis virus entry and exit and characterization of a spike protein region involved in cholesterol dependence.. J Virol.

[61] Smit JM, Klimstra WB, Ryman KD, Bittman R, Johnston RE (2001). PE2 cleavage mutants of Sindbis virus: correlation between viral infectivity and pH-dependent membrane fusion activation of the spike heterodimer.. J Virol.

[62] Sambrook J, Fritsch E, Maniatis T (1989). Molecular Cloning: a Laboratory Manual..

[63] Higgs S, Olson KE, Kamrud KI, Powers AM, Beaty BJ, Crampton JM, Beard CB, Louis C (1997). Viral expression systems and viral infections in insects.. The Molecular Biology of Disease Vectors: A Methods Manual.

[64] Vanlandingham DL, Tsetsarkin K, Klingler KA, Hong C, McElroy KL (2006). Determinants of vector specificity of o'nyong nyong and chikungunya viruses in *Anopheles* and *Aedes* mosquitoes.. Am J Trop Med Hyg.

[65] McElroy KL, Tsetsarkin KA, Vanlandingham DL, Higgs S (2006). Role of the yellow fever virus structural protein genes in viral dissemination from the *Aedes aegypti* mosquito midgut.. J Gen Virol.

[66] Pilitt DR, Jones JC (1972). A qualitative method for estimating the degree of engorgement of *Aedes aegypti* adults.. J Med Entomol.

