# Supplementary figures and images for: Epistatic Roles of E2 Glycoprotein Mutations in Adaption of Chikungunya Virus to Aedes Albopictus and Ae. Aegypti Mosquitoes
Source: PLoS One. 2009 Aug 31;4(8):e6835. doi: 10.1371/journal.pone.0006835 (PMC2729410; doi:10.1371/journal.pone.0006835)

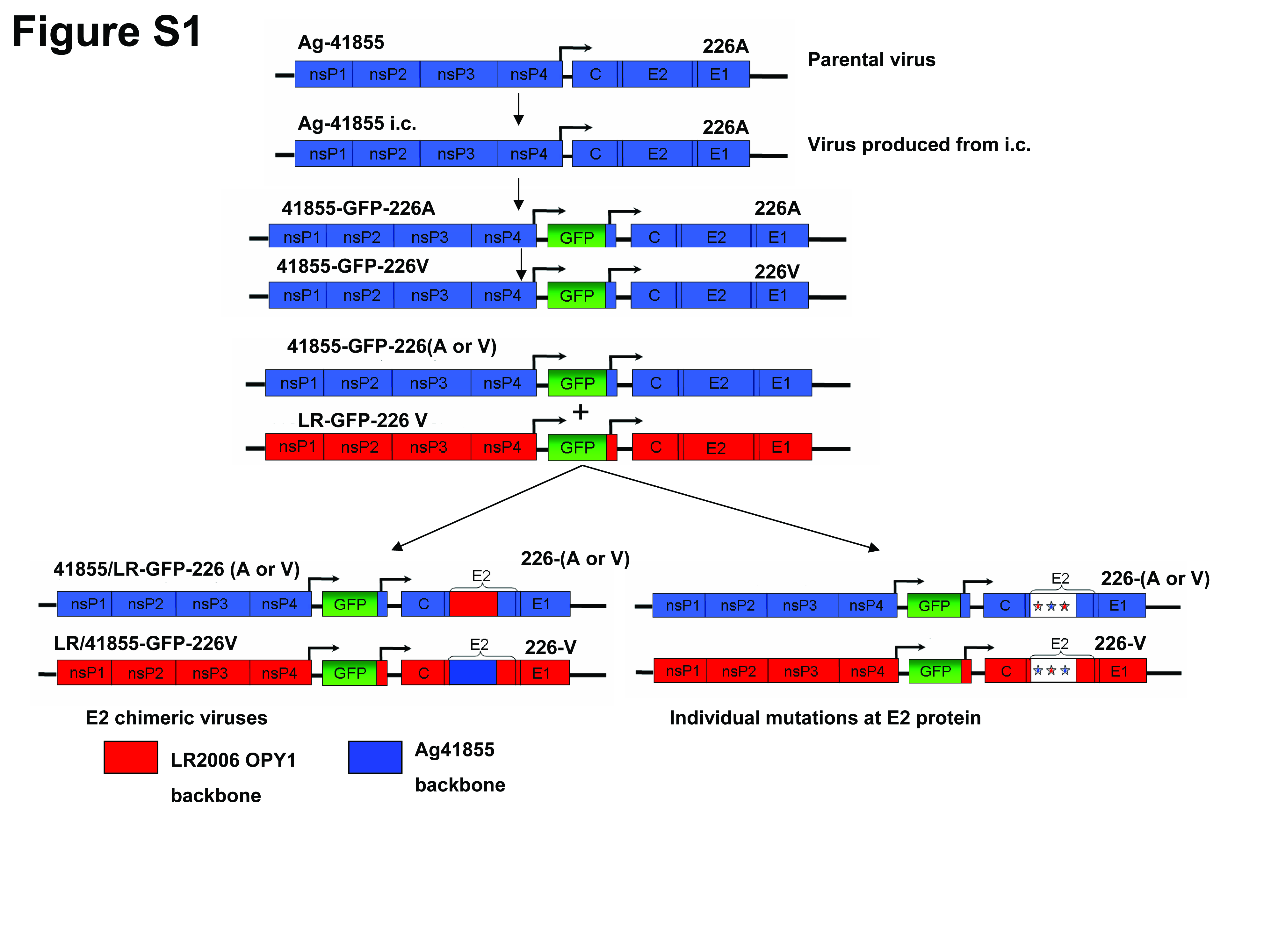

Supplement: Figure S1 — Schematic representation of the viruses used in this study. (1.20 MB TIF) [file pone.0006835.s001.tif]
