# Supplementary material for: Epistatic Roles of E2 Glycoprotein Mutations in Adaption of Chikungunya Virus to Aedes Albopictus and Ae. Aegypti Mosquitoes
Source: PLoS One. 2009 Aug 31;4(8):e6835. doi: 10.1371/journal.pone.0006835 (PMC2729410; doi:10.1371/journal.pone.0006835)
Supplement: Table S1 — Recovery of the viruses with mutations in E2 protein after electroporation of in vitro transcribed RNA. a - amino acids at position of E1-226. b - amino acids at position of E2: 60, 162, 211. c - Specific infectivity of in vitro transcribed RNA. 10(7) BHK-21 cells were transfected with 10 µg of RNA. Electroporated BHK-21 cells were ten fold serially diluted, seeded in 6 well tissue culture plates containing 1×10(6) Vero cells per well in MEM media. Following an incubation for 2 h at 37°C, cells were overlaid with 2 mL of 0.5% agarose containing 3.3% FBS in MEM. Plaques were scored and measured on day 2 post transfection. d - Supernatants of electroporated BHK-21 cells were collected on days 1 and 2. Virus titers were determined by titration on Vero cells and expressed as Log10TCID50/ml. e - Plaque size of infectious centers expressed in millimeters±standard deviation. h - hours post-infection. Blue color corresponds to authentic amino acids at indicated positions of strain Ag41855, red color corresponds to authentic amino acids at indicated positions of strain LR2006 OPY1. (0.06 MB DOC) [file pone.0006835.s002.doc]

#### Table S1. Recovery of the viruses with mutations in E2 protein after electroporation of *in vitro* transcribed RNA.

| Backbone | Clone name | E1 226 ^a^ | E2 ^b^ | | | Specific infectivity ^c^ | Titers ^d^ | | Plaque size±SD ^e^ |
| --- | --- | --- | --- | --- | --- | --- | --- | --- | --- |
|  |  |  | 60 | 162 | 211 |  | 24h | 48h |  |
| 41855-GFP | 41855-GFP-226V | V | G | V | I | 5.0x10^5^ | 8.95 | 8.52 | 3.10 ±0.29 |
|  | NG |  | D | V | I | 5.0x10^5^ | 7.95 | 7.21 | 3.00±0.82 |
|  | NG |  | G | A | I | 4.0x10^5^ | 6.95 | 7.71 | 3.17±0.24 |
|  | NG |  | G | V | T | 8.0x10^5^ | 7.95 | 7.95 | 3.41±0.41 |
|  | NG |  | G | A | T | 4.5x10^5^ | ND | 8.52 | 3.33±0.24 |
|  | NG |  | D | V | T | 9.5x10^5^ | 7.95 | 7.52 | 2.83±0.47 |
|  | NG |  | D | A | I | 7.5x10^5^ | 7.95 | 7.95 | 2.83±0.47 |
|  | 41855/LR-GFP-226V |  | D | A | T | 10x10^5^ | 7.95 | 7.95 | 3.17±0.24 |
|  | 41855-GFP-226A | A | G | V | I | 10x10^5^ | 8.52 | 7.95 | 3.33±0.29 |
|  | NG |  | D | V | I | 10x10^5^ | ND | 7.71 | 3.00±0.82 |
|  | NG |  | G | A | I | 10x10^5^ | 7.95 | 7.52 | 2.83±0.24 |
|  | NG |  | G | V | T | 8x10^5^ | ND | 7.95 | 3.00±0.82 |
|  | NG |  | G | A | T | 10x10^5^ | 8.52 | 7.95 | 3.00±0.82 |
|  | NG |  | D | V | T | 10x10^5^ | 7.95 | 7.95 | 3.00±0.41 |
|  | NG |  | D | A | I | 8.0x10^5^ | 8,52 | 7.52 | 3.33±0.24 |
|  | 41855/LR-GFP-226A |  | D | A | T | 6.8x10^5^ | ND | 7.95 | 3.10±0.29 |
