# Supplementary material for: Epistatic Roles of E2 Glycoprotein Mutations in Adaption of Chikungunya Virus to Aedes Albopictus and Ae. Aegypti Mosquitoes
Source: PLoS One. 2009 Aug 31;4(8):e6835. doi: 10.1371/journal.pone.0006835 (PMC2729410; doi:10.1371/journal.pone.0006835)
Supplement: Table S2 — Summary of virus strains used in phylogenetic analysis. Genotype: ECSA - Eastern/Central/South African; W. Afr - West African. Passage history: SM: suckling mouse; C6/36: Ae. albopictus cell line; Vero: African green monkey cell line; RMK: Rhesus monkey kidney cell line; MRC-5: human lung epithelium; AP61: Ae. pseudoscutellaris cell line. GenBank Acc - GenBank accession number. ? - information is unavailable to the authors (0.10 MB DOC) [file pone.0006835.s003.doc]

#### Table S2. Viruses used in phylogenetic analysis.

| Name  in phylogeny | Strain Name | Genotype | Location of isolation | Year of isolation | Source of isolate | Passage history | E2-60 | E2-211 | GenBank Acc |
| --- | --- | --- | --- | --- | --- | --- | --- | --- | --- |
| CAR80v1 | DakAr B 16878 | ECSA | Central African Region (*Bouboui*) | 1980 | *An. funestus* | SM-5; Vero-2; C6/36-1 | D | I |  |
| CARb86v10 | CAR256 | ECSA | Central African Region | (before 1986) | ? | SM-1; (Yaru); C6/36-1 | D | I |  |
| DRC60v29 | LSF5 | ECSA | Democratic Republic of Congo | 1960? | human | SM1; Vero1; C6/36-1 | D | I |  |
| LR2006 OPY1 | LR2006 OPY1 | ECSA | France (*imported from La Reunion*) | 2006 (17 Feb) | human | Vero-5; SM-1; C6/36-1 | D | T | DQ443544 |
| GAB 07 | Gabon 2007 | ECSA | Gabon | 2007 | ? | ? | D | T |  |
| GERb07 | Wuerzburg-1 | ECSA | Germany (*imported from Mauritius*) | (before Sept 2007) | human | ? | D | T | EU037962 |
| INDI00 | IND-00-MH4 | ECSA | India (*Yawat, Maharashtra - western*) | 2000 | mosquito | C6/36-3; SM-3 | D | I | EF027139 |
| INDI06ap | IND-06-AP3 | Asian | India (*Andhra Pradesh*) | 2006 early | human | C6/36-1 | D | T | EF027134 |
| INDI06ka | IND-06-KA15 | Asian | India (*Karnataka*) | 2006 early | human | C6/36-2 | D | T | EF027135 |
| INDI06rj | IND-06-RJ1 | Asian | India (*Rajasthan*) | 2006 late | human | C6/36-2 | D | T | EF027137 |
| INDI63 | IND-63-WB1 | Asian | India (*Kolkata, West Bengal - eastern*) | 1963 | human | SM-6; C6/36-1 | D | T | EF027140 |
| INDI73 | IND-73-MH5 | Asian | India (*Barsi, Maharashtra*) | 1973 | human | SM-1; C6/36-1 | D | T | EF027141 |
| INDO85v15 | RSU1 (TVP1336) | Asian | Indonesia (*Maluku, Ambon Island*) | 1985 | human | Vero-2; C6/36-1 | D | T |  |
| ITA07 | ITA07-RA1 | ECSA | Italy (*Ravenna*) | 2007 | ? | ? | D | T | EU244823 |
| MAU06 | D570/06 | ECSA | Mauritius | 2006 (March) | human | Vero-1 | D | T | EF012359 |
| NIG64v3 | IbH35 | W. Afr. | Nigeria (*Ibadan*) | 1964 | human | SM?; Vero-1; C6/36-1 | D | T |  |
| NIG65v26 | IbAn4824 | W. Afr | Nigeria | 1965 (13 Apr) | sentinel mouse brain | (NH) SM2; C6/36-1 | G | T |  |
| PHI85v18 | Hu/85/NR/001 | Asian | Philippines | 1985 | human | Vero-2 | D | T |  |
| REU05csf | 06-027 | ECSA | La Reunion (*South, St Joseph*) | 2005 (29 Nov) | human | C6/36-1 to 2 | D | T | AM258993 |
| REU05dec | 06-049 | ECSA | La Reunion (*South, St. Louis*) | 2005 (2 Dec) | human | C6/36-1 to 2 | D | T | AM258994 |
| REU05may | 05-115 | ECSA | La Reunion (*South, La Riviere St Louis*) | 2005 (6 May) | human | C6/36-1 to 2 | D | T | AM258990 |
| REU05nov | 06-021 | ECSA | La Reunion (*South, La Riviere St Louis*) | 2005 (28 Nov) | human | C6/36-1 to 2 | D | T | AM258992 |
| SAF56v9 | Verseeniging | ECSA | South African Republic | 1956 (April) | human | SM-9; Vero-1; C6/36-1 | G | I |  |
| SAF76v13 | SAH2123 | ECSA | South African Republic | 1976 | human | Mosquito-1; SM-2; C6/36-1 | D | T |  |
| SAF76v24 | AR 18211 | ECSA | South African Republic | 1976 | *Ae. furcifer* | Mosquito-2; SM-3; C6/36-1 | D | T |  |
| SEN66v2 | PM2951 | W. Afr | Senegal (*Ndofore*) | 1966 (Nov) | *Ae. aegypti* | Sm3; Vero-1; C6/36-1 | D | T |  |
| SEN83 | 37997 | W. Afr | Senegal (*Kedougou*) | 1983 (10 Oct) | *Ae. furcifer* | AP61-1; Vero-2; C6/36-1 | D | T | AY726732 |
| SENb81v23 | IPD/A SH 2807 | W. Afr | Senegal | (before 1981) | human | SM-3; mosquito-1; C6/36-1 | D | T |  |
| SEY05 | 05-209 | ECSA | Seychelles (*Mahe Island, Anse aux Pins*) | 2005 (9 Aug) | human | C6/36-1 to 4 | D | T | AM258991 |
| SLA07v20 | SL-CK1 | ECSA | Sri Lanka | 2007 | human | Vero-1; C6/36-1 | D | T |  |
| TAN53a | Ross (*from GenBank*) | ECSA | Tanzania (*Liteho, Newala District*) | 1953 (22 Feb) | human | SM-176; Vero-2 | D | I | AF490259 |
| TAN53b | S27 (*from GenBank*) | ECSA | Tanzania (*Liteho, Newala District*) | 1953 (22 Feb) | human | (unspecified high-passage) | D | I | AF369024 |
| TAN53v32 | S27 | ECSA | Tanzania (*Liteho, Newala District*) | 1953 (22 Feb) | human | SM175; Vero1; C6/36-1 | D | I |  |
| TAN53v33 | Ross (*low-psg*) | ECSA | Tanzania (*Liteho, Newala District*) | 1953 (22 Feb) | human | SM16; Vero1; C6/36-1 | G | I |  |
| THAI58v30 | TH35 | ECSA | Thailand (*Bangkok*) | 1958 | human | SM21; C6/36-1 | D | T |  |
| THAI62 | AF15561 | Asian | Thailand (*Bangkok*) | 1962 | human | Vero-2; | D | T | EF452493 |
| THAI95v14 | CO392-95 | Asian | Thailand (*Bangkok*) | 1995 | human | LLC-MK2-1; Vero-1; C6/36-1 | D | T |  |
| Ag41855 | Ag41855 | ECSA | Uganda (*Mukono District*) | 1982 | human | SM3; Vero1 | G | I |  |
